# Superionic Germanium Substituted Lithium Thioarsante Li_6+_ *_x_* Ge*_x_* As_1‐_ *_x_* S_5_Br Argyrodites With High Air Stability

**DOI:** 10.1002/smll.74170

**Published:** 2026-07-30

**Authors:** Ajay Gautam, Ruihua Zhou, Anastasia K. Lavrinenko, Hanan Al‐Kutubi, Swapna Ganapathy, Xin Zhang, Alexandros Vasileiadis, Shuo Wang, Marnix Wagemaker

**Affiliations:** ^1^ Storage of Electrochemical Energy Department of Radiation Science and Technology Faculty of Applied Sciences Delft University of Technology Delft The Netherlands; ^2^ Materials Engineering Indian Institute of Technology Jammu Jammu Jammu and Kashmir India; ^3^ State Key Laboratory of Advanced Glass Materials School of Material Science and Engineering Wuhan University of Technology Wuhan China

**Keywords:** air stability, ionic conductivity, jump local environments, solid electrolytes, structure‐transport correlations

## Abstract

Lithium argyrodite solid electrolytes are promising for all‐solid‐state batteries due to their high ionic conductivity and low elastic modulus. However, poor chemical stability in humid conditions remains a major hurdle towards large‐scale practical implementation. This motivates substituting phosphorus with softer acids to stabilize sulfur, where the challenge is to maintain high Li‐ion mobility. In this work, we explore the effects of aliovalent (Ge) substitutions on the structure, ionic transport, and air stability of Li_6_AsS_5_Br. The induced structural modification releases the rate‐limiting step in long‐range Li‐ion transport, as demonstrated by Molecular Dynamics and percolation simulations. As a result, the ionic conductivity reaches 13 mS/cm for Li_6.5_As_0.5_Ge_0.5_S_5_Br, a 6‐fold improvement over pristine Li_6_AsS_5_Br. The key enabler for combining high Li‐ion mobility and improved stability towards moisture is the increased polarity of the Ge─S bonds compared to As─S or P─S bonds. This reduces Li‐ion trapping effectively flattening the energy landscape for diffusion and thermodynamically suppresses H_2_S formation upon exposure to moisture, as experimentally demonstrated. Finally, stable cycling performances in combination with high nickel ternary cathodes are demonstrated. Hereby, this research provides a deeper understanding of the role of composition on the ionic conductivity and moisture stability of lithium argyrodites.

## Graphical abstract

Ge substitution in Li_6+__x_Ge_x_As_1‐x_S_5_Br expands the lattice, redistributes Li^+^, and enhances intercage diffusion, increasing ionic conductivity 6‐fold to 12.7 mS/cm at *x* = 0.5. Li_6.5_As_0.5_Ge_0.5_S_5_Br also shows improved air stability, linking the lithium substructure to fast, stable ion transport in thioarsenate argyrodites.

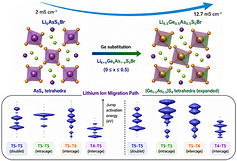

## 1 Introduction

Lithium‐ion batteries (LIBs) have become pivotal in powering portable electronics, electric vehicles, and storing renewable energy, which may help to reduce the carbonization of the environment [1, 2, 3]. Current LIB technology exhibits good electrochemical performance and durability [4, 5]. Despite their advantages, LIBs' current use of liquid electrolytes raises significant safety concerns, including risks of flammability, leakage, and dendrite formation with a lithium metal anode, which can compromise battery integrity and longevity [6]. Solid‐state batteries (SSBs), which use solid electrolytes, offer a safer and potentially more efficient alternative as the next‐generation energy technology [7, 8]. They promise higher energy densities with high‐capacity anodes such as Li/ Si, enable the high voltage‐cathode [9, 10], and operate in wider temperature ranges [11]. To further improve the performance, solid electrolytes should exhibit high ionic conductivity, ease of processability, and robust chemical stability [12, 13, 14]. Various families of solid electrolytes have been explored, including lithium halides [15, 16], oxides [17, 18], lithium thiophosphates [19, 20, 21, 22, 23], antiperovskites [24], and lithium argyrodites [25, 26, 27, 28]. Among these, lithium argyrodites, particularly Li_6‐x_PS_5_X_1+x_ (X = Cl, Br), have gained significant attention due to their high ionic conductivity, mechanical softness, and straightforward synthesis [26, 29, 30, 31, 32].

The lithium argyrodite Li_6_AsS_5_Br has a cubic structure with space group $F\bar{4}3m$, where halide ions are positioned in a cubic lattice at the 4a Wyckoff position and sulfur ions are distributed over the tetrahedral Wyckoff 4*d* and 16*e* sites [28]. An exchange of Br^−^ and S^2−^ at the 4*d* site can occur, known as site‐disorder [33, 34]. The P^5+^ cation occupies the octahedral 4*b* site (PS_4_^3−^ tetrahedra with S at the 16*e* site). The 132 remaining interstitial voids can be occupied by lithium in different configurations, such as lithium tetrahedra Li(S/X)_4_, which connect via face, edge, or corner with the PS_4_^3−^ tetrahedra, designated as T1‐T5a [35], as depicted in Figure S1. Hereby, lithium forms cage‐like structures around the Wyckoff 4*d* site, and can additionally partially occupy various other crystallographic sites, promoting fast ion diffusion. The lithium argyrodite Li_6_PS_5_I was initially reported as a poorly conducting material due to its lack of site disorder. Aliovalent substitution, for instance replacing P with lower‐valence cations (Si, Ge, In, and Sn) [36] and isovalent substitutions, including substitution of P (As, and Sb) [28, 37], S (O, and Se) [38, 39, 40, 41], and halide substitution/enrichment (Cl, Br, and I) [31, 42, 43, 44] has been employed to alter the crystal structure, targeting enhanced lithium ion transport [42, 43, 44, 45]. For example, aliovalent substitution in iodide argyrodite, replacing P^5+^ with Ge^4+^ in Li_6−x_P_1‐x_Ge_x_S_5−x_I, resulted in ionic conductivities up to 5.4 mS/cm in cold‐pressed pellets [46, 47, 48]. Likewise, novel thioantimonate argyrodites with Si^4+^ and Ge^4+^ replacing Sb^5+^ in Li_6+x_Sb_1‐x_M_x_S_5−x_I (M = Si, Ge) exhibit ionic conductivities reaching 14.8 mS/cm in cold‐pressed pellets [49]. Aliovalent substitutions in Li(P/Sb)_1‐x_M_x_S_5_I (M = Si, Ge, and Sn) with 10–30 wt.% substitution have led to a sharp increase in ionic conductivity from the µS/cm to the mS/cm range, accompanied by a reduction in activation energy from 0.38 to 0.24 eV [46]. This improvement is potentially linked to increased polyhedral volume, higher lithium content enabling inter‐cage jumps for long‐range diffusion, and site disorder influencing lithium distribution. However, these aspects of the material have not yet been fully explored and require further investigation.

Lithium thiophosphate‐based materials suffer from poor air and moisture stability, reacting with H_2_O to release harmful H_2_S gas and experiencing rapid structural degradation and performance reduction, posing challenges in their large‐scale application in SSBs. According to the Hard Soft Acid Base (HSAB) theory, substituting phosphorus (P) with softer acids such as germanium (Ge), arsenic (As), or antimony (Sb) could enhance the moisture stability of these solid electrolytes [50, 51]. Several P‐free thio‐LISICON structures (Li_4‐x_Ge_1‐x_As_x_S_4_ [50], Li_4‐x_Sn_1‐x_As_x_S_4_ [51], and Li_4‐x_Sn_1‐x_Sb_x_S_4_ [52]) have been developed, indeed demonstrating improved air stability [50, 51]. Minafra et al. reported that substituting 30% of P with Si in Li_6_PS_5_Br is possible, resulting in a 3‐fold improvement in ionic conductivity [37]. Recent studies have demonstrated that only up to 0.35 wt.% Ge can be substituted in the Li_6_PS_5_Br argyrodite series, resulting in a 5‐fold increase in ionic conductivity [48]. However, further substitution is not feasible due to the relatively large ionic radius of Ge^4^^+^. In our previous work, we replaced P^5^^+^ (17 pm) with the larger As^5^^+^ ion (33.5 pm) in Li_6_P_1‐x_As_x_S_5_Br, which led to an expansion of the lattice parameters. This structural expansion may enable higher Ge^4^^+^ incorporation, thereby motivating further investigation into the lithium substructure and its impact on ionic transport.

In this work, we investigate the effects of Ge substitution on the crystal structure, ionic transport, and air stability of Li_6_AsS_5_Br. We successfully synthesized a series of samples and characterized their crystal structures through Rietveld refinement of neutron diffraction, solid‐state NMR, and impedance spectroscopy. Our analysis reveals that Ge substitution not only enlarges the polyhedral volume of (As^5+^/Ge^4+^)S_4_^3/4−^ but also increases the lattice parameters and lithium content, thereby ensuring a more uniform distribution of lithium within the cages. Moreover, increased lithium occupation at the Li T4 site flattens the energy landscape for lithium diffusion, facilitating fast ion transport. The highest ionic conductivity achieved is 13 mS/cm, for the Li_6.5_As_0.5_Ge_0.5_S_5_Br composition, which is a 6‐fold increase compared to pristine Li_6_AsS_5_Br. In addition, we employed computational approaches to elucidate the atomic‐scale mechanisms driving the observed improvements in ionic transport. Ab initio molecular dynamics (AIMD) simulations allow tracking mean square displacements (MSDs) of lithium ions in representative supercells of As/Ge‐substituted systems, providing insight into the Li‐ion diffusion mechanism. Lithium density plots reveal the preferred diffusion pathways and the degree of interconnectivity between lithium cages in different substitutional cases. Radial distribution function (RDF) analysis and cage radius measurements highlight the structural modifications influencing lithium‐ion transport. By mapping the local environments, we evaluate the accessibility of various jump types that can facilitate percolation throughout the crystal structure, explaining the fundamental mechanisms underlying the improved diffusion behavior upon introducing Ge. As anticipated based on Hard Soft Acid Base (HSAB) theory, Li_6.5_As_0.5_Ge_0.5_S_5_Br improves the air stability compared to the Li_6_(P/As)S_5_Br, suppressing H_2_S formation and improving conductivity retention upon air exposure. Hereby, this study highlights the opportunities of compositional adjustments to improve the ionic conductivity of lithium argyrodite electrolytes as well as their air stability.

## 2 Result and Discussion

**FIGURE 1:**
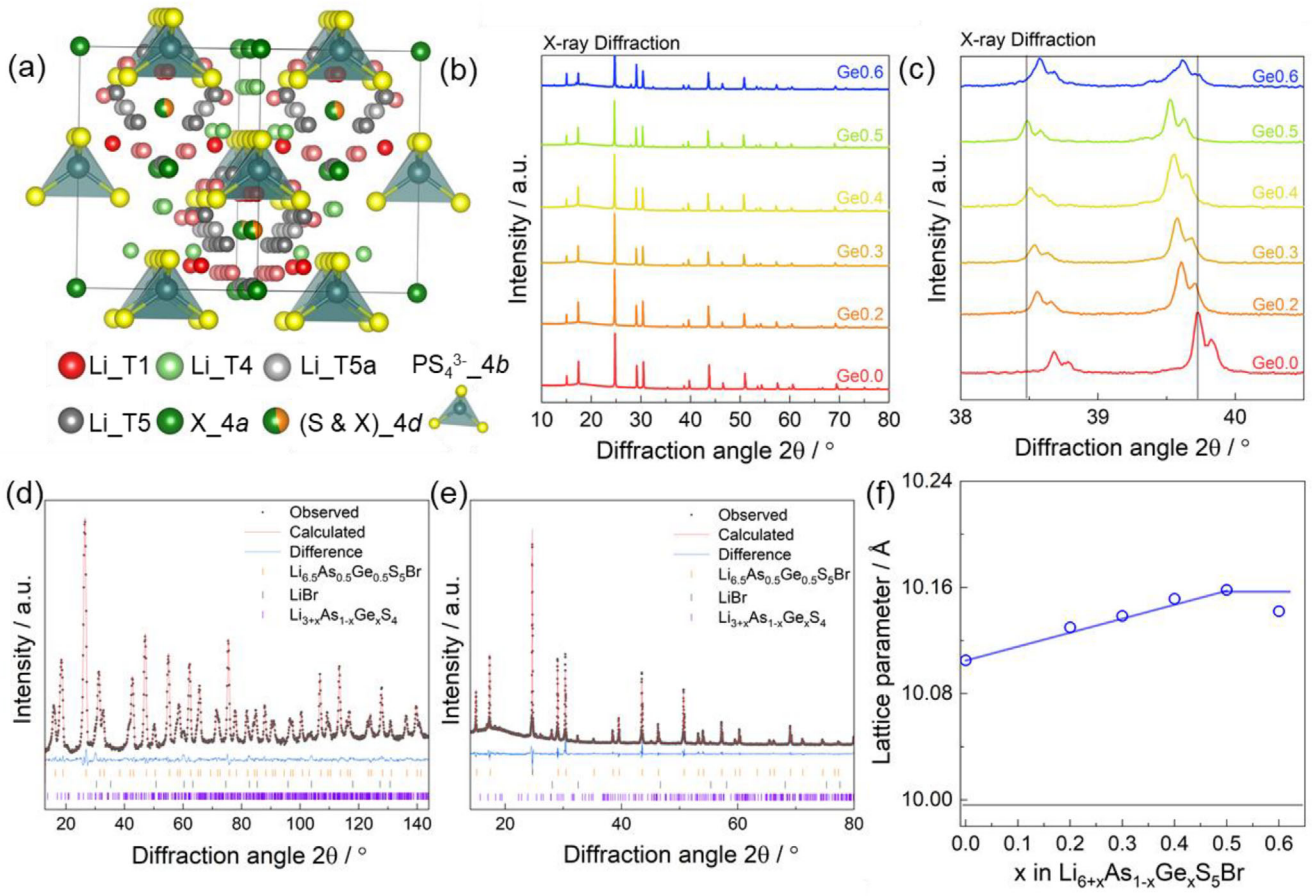
(a) Structure of Li_6_AsS_5_Br argyrodite with space group $F\bar{4}3m$, where X*^−^* and S*^2−^* occupy the Wyckoff 4d and 4a site, and the PS_4_^3−^ tetrahedral unit on Wyckoff 4b site. In the argyrodite structure, available voids are potentially occupied by lithium, forming lithium tetrahedra Li(S/X)_4_, which connect (‐face, edge, corner) with the PS_4_^3−^ tetrahedra. Lithium forms a cage‐like arrangement around the Wyckoff 4d site. (b) X‐ray diffraction patterns of the Li_6+x_As_1−x_Ge_x_S_5_Br series (c) enlarged view of the 2θ range 38 to 41° highlighting changes in lattice parameter due to Ge substitution from *x* = 0 to *x* = 0.5 in the Li_6+x_As_1−x_Ge_x_S_5_Br series. Rietveld refinement analysis of (d) neutron diffraction and (e) x‐ray diffraction data for Li_6.5_As_0.5_Ge_0.5_S_5_Br. The experimental data is represented by black dots, the calculated pattern is shown as red lines, and the blue lines depict the difference profile between the experimental and calculated data. The orange, dark brown, and purple tick marks indicate the expected peak positions of Li_6.5_As_0.5_Ge_0.5_S_5_Br, LiBr, and Li_3+x_As_1‐x_Ge_x_S_4,_ respectively. (f) Lattice parameters of the Li_6+x_As_1−x_Ge_x_S_5_Br series with Ge substitution ranging from *x* = 0 to *x* = 0.6, as calculated from Rietveld refinement of x‐ray and neutron diffraction data. The grey color line represents the lattice parameter of Li_6_PS_5_Br. Error bars are not shown because they are smaller than the symbols in the data.

A series of thioarsante solid solutions, Li_6+x_As_1−x_Ge_x_S_5_Br (x ≤ 0.6) were synthesized via a solid‐state reaction (see supporting information experimental section). For structural characterization, neutron diffraction and x‐ray diffraction (XRD) were performed, and the data were analyzed using Rietveld refinement to investigate structural changes and Li^+^ substructures. All x‐ray diffraction patterns of the Li_6+x_As_1−x_Ge_x_S_5_Br series are presented in Figure 1b. The XRD patterns show the reflections of the cubic structure of the $F\bar{4}3m$ space group. A gradual shift to lower 2θ values with increasing Ge content suggests a larger lattice parameter, as illustrated in Figure 1c. Minor LiBr impurities (0.5 wt.% LiBr, 2.3 wt.% Li_4‐x_Ge_1‐x_As_x_S_4_, for *x* = 0.4 and 2 wt.% LiBr and 5 wt % Li_4‐x_Ge_1‐x_As_x_S_4_ for *x* = 0.5) were observed, not significantly affecting ionic conductivity. However, increasing Ge^4+^ content (*x* = 0.6), a higher impurity content was observed, amounting 2 wt.% LiBr and 14 wt.% Li_4‐x_Ge_1‐x_As_x_S_4_, indicating the solubility limit of Ge in the thioarsenate series is reached. Rietveld refinements of neutron diffraction and x‐ray patterns of Li_6.5_As_0.5_Ge_0.5_S_5_Br are shown in Figure 1d,e. Details of all other refinement figures and tables are available in supporting information in Figures S2–S6 and Tables S1–S6.

Figure 1f shows that the lattice parameter of Li_6_PS_5_Br composition (grey line) is 9.98 Å. Replacing P^5+^ (17 pm) with As^5+^ increases the lattice parameter from 9.98 Å to 10.105 Å (a difference of ∼0.12 Å) for *x* = 0. As the Ge^4+^ content increases, the lattice parameter expands linearly due to the larger ionic radius of Ge^4+^ (39 pm) compared to As^5+^ (33.5 pm), also increasing the lithium content, following Vegard's rule until saturation, *x* = 0.5, while maintaining the cubic structure. The lattice parameter in the thioarsenate series expands from 10.105 Å at *x* = 0.0 to 10.158 Å at *x* = 0.5, and decreases at *x* = 0.6, along with the increasing presence of LiBr and Li_4‐x_Ge_1‐x_As_x_S_4_, impurities, suggesting that the solubility limit has been reached. The morphology and elemental distribution of the electrolyte particles were determined through the use of scanning electron microscopy with energy‐dispersive x‐ray spectroscopy (EDX) (Figure S7). The average particle size of the Li_6.5_As_0.5_Ge_0.5_S_5_Br, Li_6_AsS_5_Br, Li_5.5_PS_4.5_Br_1.5_, and Li_6_PS_5_Br samples is approximately 5 µm. The EDX results reveal that each element is distributed homogeneously at the micrometre level, demonstrating the absence of any discernible impurities.

**FIGURE 2:**
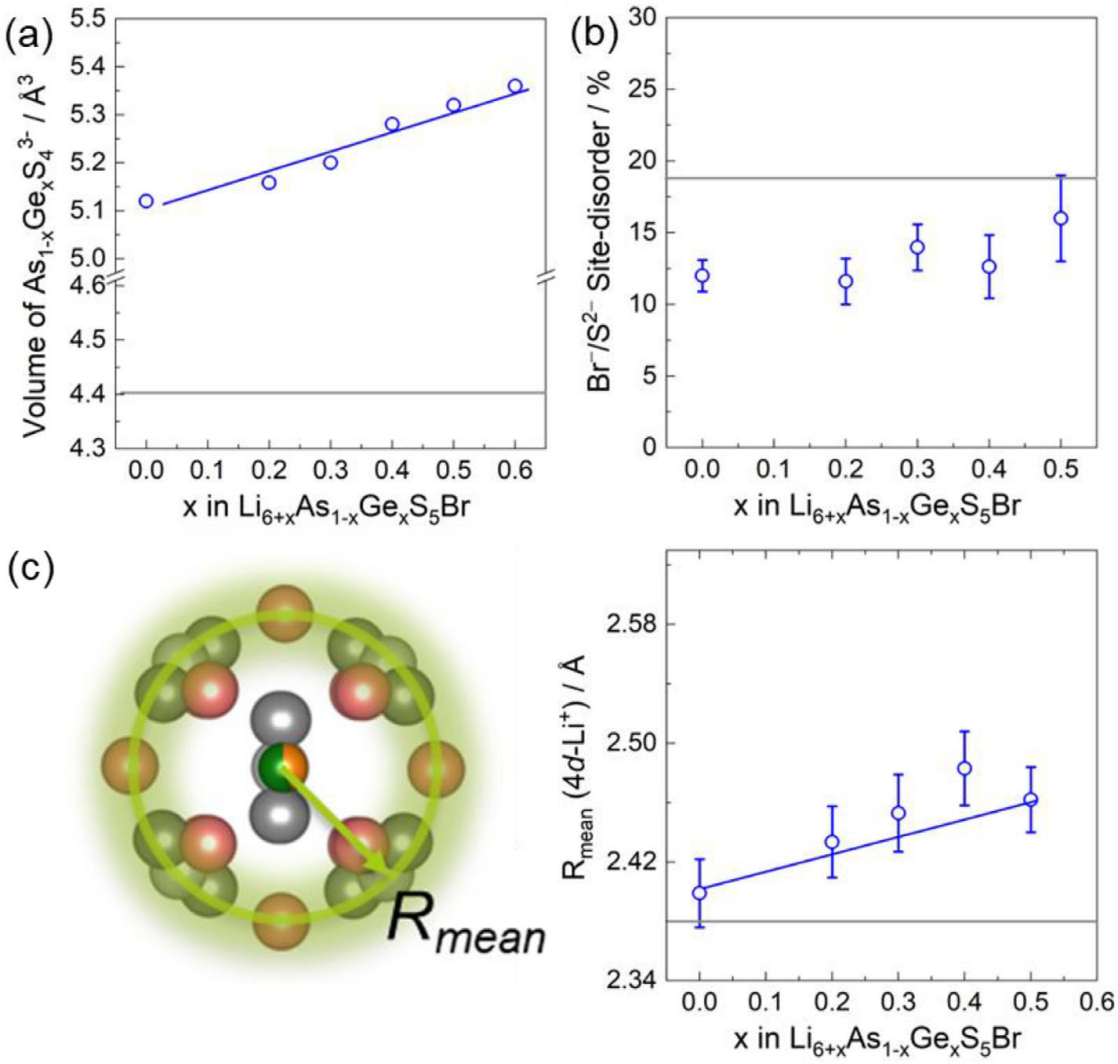
Structural change of Li_6+x_As_1−x_Ge_x_S_5_Br series calculated from Rietveld refinement of neutron diffraction data. (a) Tetrahedral volume (Ge^4+^/As^5+^) and (b) site‐disorder with increasing Ge^4+^ content in Li_6+x_As_1−x_Ge_x_S_5_Br series. (c) The visual representation of the average Li^+^ distribution, R_mean_, represents the radius of the sphere formed by Li positions centered around the anion position (Wyckoff 4d). (d) As the Ge content increases, R_mean_ increases, representing an expansion of the Li^+^ cages, resultant decreasing inter‐cage jump distance. The grey color line represents the (a) Tetrahedral volume PS_4_^3−^, (b) Site‐disorder, (d) R_mean_ value of Li_6_PS_5_Br.

The thiophosphate series exhibits a polyhedral volume of 4.41 Å^3^ (Li_6_PS_5_Br), which increases to 5.05 Å^3^ in the thioarsenate (Li_6_AsS_5_Br) due to the larger ionic radius of As^5+^ (33.5 pm) compared to P^5+^ (17 pm). As the Ge content increases, the polyhedral volume of (Ge^4+^/As^5+^)S_4_^(3+x)−^ expands further from 5.05 Å^3^ at *x* = 0 to 5.35 Å^3^ at *x* = 0.5, attributed to the higher ionic radius of Ge (39 pm). This expansion confirms successful aliovalent substitution, as illustrated in Figure 2a. In the Li_6_PS_5_Br composition, 18% site‐disorder is observed, while Li_6_AsS_5_Br found 12% site‐disorder. As Ge^4+^ content increases, site disorder fluctuates between 12% and 15%, as demonstrated in Figure 2b. However, there appears to be no distinct correlation between the degree of site‐disorder and the Ge^4+^ content.

A previous study has found that Li partially occupied different crystallographic sites, offering a 3D framework for fast lithium long‐range jumps within the unit cell [45]. De Klerk et al., using DFT simulations, suggested that higher ionic conductivity is achieved at around 75% site disorder, as this balances the different lithium‐ion jump pathways: doublet (through the nominal T5a transition site), intra‐cage (between two adjacent T5 tetrahedra), and inter‐cage jumps (via adjacent T5 sites) [53]. A recent study has further identified a new jump that contributes to lithium‐ion jump pathways: inter‐cage jumps (occurring first via adjacent T2 sites and then through T5 sites via the T4 site) [35], as shown in Figure S8. Lithium argyrodite, where the Li‐ion only occupies the T5 and T5a Wyckoff sites, usually has poor Li‐ion conductivity due to a large intra‐cage jump distance [53]. Our previous study determined that Br‐rich thioarsante possesses a shortened intercage jump distance, facilitating higher ion diffusivity. To understand the lithium substructure of the Li_6+x_As_1−x_Ge_x_S_5_Br series, we employed neutron powder diffraction, analysed by Rietveld refinement. Initial structural information, such as cation/anion position and occupancy, was taken from the Li_6_PS_5_Br composition as a reference [45]. The overall Li^+^ content was adjusted to maintain charge balance with the refined Ge content. Figure S9 shows the lithium site occupancies (T5, T2, and T4) across the thioarsenate argyrodite series, as determined by Rietveld refinement, revealing composition‐dependent variations at each site.

Figure S10 illustrates the lithium volume at T2, T4, and T5 Li sites as a function of Ge content. Increasing Ge content not only enlarges the tetrahedral volume but also affects the surrounding Li^+^ substructure. The volume of T2 and T5 sites increases, following a trend similar to the polyhedral volume of (Ge^4+^/As^5+^)S_4_^(3+x)−^, while the volume of the T4 site remains largely unchanged despite the increased Ge content. Figure S11 displays the T5−T2−T2−T5 and T5−T4−T5 distances as a function of Ge content, noting that the T5−T2−T2−T5 distances decrease from 5.01 (4) Å at *x* = 0.0 to 4.51 (8) Å at *x* = 0.5, As mentioned above, the inter‐cage connectivity between lithium cages is essential for long‐range Li^+^ diffusion. Additionally, the T5−T4−T5 distance remains the same, but the Li T4 occupancies increase from 1.0 (5)% at *x* = 0.0 and 6.0 (9)% at *x* = 0.5, potentially increasing the connectivity between lithium cages due to lower energy inter‐cage jumps [54]. To calculate the Li^+^ distributions (R_mean_) across all sites as determined by Rietveld refinement. A graphical representation of R_mean_ is presented in Figure 2c. As the Ge content increases in the thioarsenate argyrodite series, there is a concomitant increase in R_mean_ values (as shown in Figure 2d). This can be attributed to lattice expansion, increased tetrahedral volumes, and higher lithium content, which together enhance cage connectivity and thereby promote improved ionic transport.

### 2.1 Ionic Transport

**FIGURE 3:**
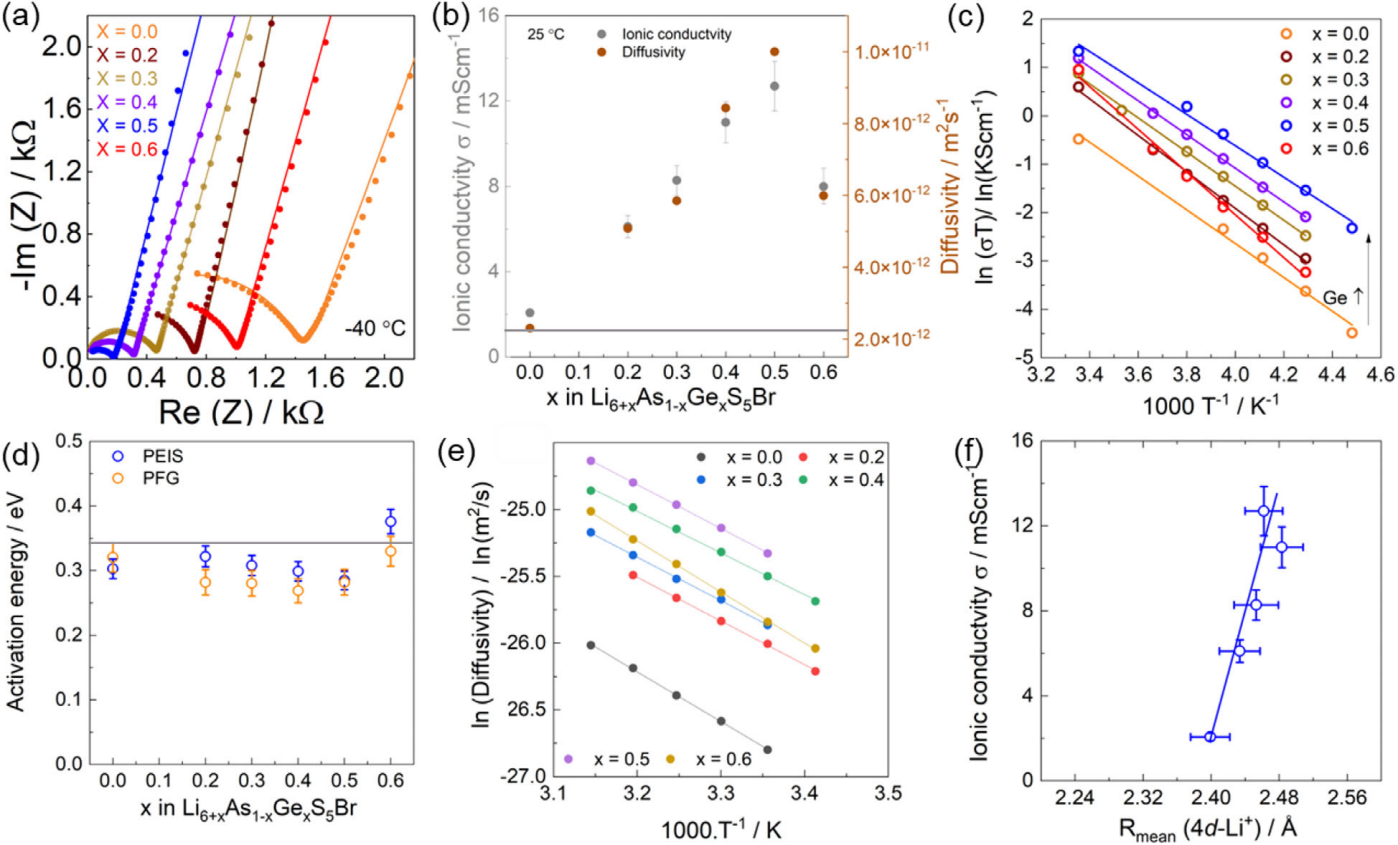
(a) Impedance spectra of the Li_6+x_Ge_x_As_1−x_S_5_Br series at −40°C. (b) Ionic conductivities and Li‐ion diffusion coefficient of Li_6+x_Ge_x_As_1−x_S_5_Br series at room temperature, measured by impedance spectroscopy and pulsed field gradient NMR respectively. (c) Represents Arrhenius plots, obtained from temperature‐dependent impedance spectroscopy. and (d) activation energy E_A_ of Li_6+x_Ge_x_As_1−x_S_5_Br as a function of the Ge content. (e) Temperature dependent Li^+^ ion diffusivity obtained from experimental ^7^Li PFG NMR measurements of Li_6+x_Ge_x_As_1−x_S_5_Br series. (f) The room‐temperature ionic conductivity is shown as a function of the R_mean_ distance.

Temperature‐dependent electrochemical impedance spectroscopy (EIS) was performed to calculate the total ionic conductivity and activation energy of Li_6+x_Ge_x_As_1−x_S_5_Br series, as depicted in Figure 3. The temperature varied from −50°C to −10°C with increments of 10°C, with an additional point at 25°C. Figure S12 displays impedance data at lower temperatures, from −50°C to −10°C. Impedance data were modelled using an equivalent circuit composed of a resistor and a constant phase element (CPE) in parallel, followed by another CPE in series, representing the blocking behavior of the steel electrode. At higher temperatures, the contribution from the resistor/CPE configuration shifted to higher frequencies beyond the measurable range of the instrument. Hence, we only utilized the tail of the impedance data for the impedance fitting, modelled with a Resistor and CPE in series. Figure 3a displays the Nyquist plots for the Li_6+x_Ge_x_As_1−x_S_5_Br series at −40°C with varying compositions (*x* = 0.1 to 0.6). Notably, as the Ge^4+^ content increases, the resistance of the materials decreases, indicative of enhanced ionic conductivity. The obtained capacitances range from 2 × 10^−10^ to 2.6 × 10^−10^ F. The bulk and grain boundary effects cannot be deconvoluted, and the ideality factors (α) span from 0.80 to 0.86, representing only a single semicircle, which overall represents ionic conductivities. Figure 3b illustrates room‐temperature ionic conductivities of the Li_6+x_Ge_x_As_1−x_S_5_Br series with Ge‐content, alongside reference values from Li_6_PS_5_Br for comparison [45]. Ionic conductivity increases from 2.07 mS/cm at *x* = 0.0 to 12.7 mS/cm at *x* = 0.5 in the Li_6+x_Ge_x_As_1−x_S_5_Br series, which is the highest ionic conductivity obtained in the lithium argyrodites series prepared by cold pressing to date. As mentioned above, Ge substitution of *x* = 0.6 exceeds the solubility limit, leading to a slight reduction in conductivity due to increased impurity phases. Figure 3c reveals that the temperature‐dependent ionic conductivities follow an Arrhenius trend, allowing for the calculation of the activation energy. Interestingly, Figure 3d shows only a small difference in the activation energies with Ge^4+^ substitution, the activation energy ranging from 0.303 eV at *x* = 0 to 0.285 eV at *x* = 0.5 in the thioarsante series.

Figure S13 demonstrates that carrier charge density does not directly correlate with ionic conductivity. At this stage, the ionic conductivities are primarily attributed to the expansion of the Li^+^ cages and the consequential reduction in the inter‐cage jump distance, which is considered rate‐limiting for the bulk conductivity in argyrodites. The Li_6‐x_As_5‐x_Br_1+x_ (0 ≤ x ≤0.5) Br‐enriched compositions exhibit higher Br content at the 4*d* site and lithium deficiency, which will affect the electrostatic interactions between Li^+^ and S^2−^ ions [30]. However, in the Li_6+x_Ge_x_As_1−x_S_5_Br series, changes in polyhedral volumes, expanding the lithium substructure and effecting the electrostatic interactions between Li^+^ and the GeS_4_^4–^ polyanion, and larger Li concentration can be anticipated to the improved could make more connections between the lithium cages playing a crucial role in improving conductivity.

The ionic conductivity, as determined by Electrochemical Impedance Spectroscopy (EIS), is influenced by contributions from grain boundaries and applied pressure. These factors often lead to variations in conductivity values observed across different laboratories while studying the same solid electrolyte composition [55]. Pulsed Field Gradient NMR (PFG NMR) can provide a more direct technique to determine the bulk diffusivity, and gain more trust in the Li‐ion mobility and conductivity values. ^7^Li PFG NMR spectroscopy was conducted between 25°C and 80°C. Analysis of spectra, which depicted echo intensity against gradient strength, utilized a single‐component fit. Benchmark diffusion coefficients for solid ion conductors are as follows: Li_10_GeP_2_S_12_ at 2.2 × 10^−12^ m^2^/s, Li_10_SiP_2_S_12_ at 3.5 × 10^−12^ m^2^/s or Li_5.5_PS_4.5_Cl_1.5_ at 1.01 × 10^−11^ m^2^/s [43, 56, 57]. Figure 3b shows the room temperature, the diffusion coefficient for Li_6_AsS_5_Br at 2.3 × 10^−12^ (*x* = 0.0), which increases to 11.6 × 10^−11^ m^2^/s in Li_6.5_As_0.5_Ge_0.5_S_5_Br (*x* = 0.5), reflecting a trend similar to the improvements observed in ionic conductivity by EIS. The diffusion coefficient from PFG NMR is a measure of the mobility of the ions in a material, driven by thermal motion, thus quantifying the bulk diffusivity. Similar to the ionic conductivity, the lithium diffusion coefficient also increases by a factor of 5–6 for the Li_6+x_Ge_x_As_1−x_S_5_Br series, as shown in Figure 3b. Additionally, the ionic conductivity was calculated from the PFG‐NMR diffusion coefficient using the Nernst‐Einstein equation σ_NMR_ = cq^2^D*/K_B_T, where D* is the Li self‐diffusion coefficient obtained from PFG‐NMR, q is the elementary charge of Li^+^, k_B_ is the Boltzmann constant, T is the temperature, and c is the number density of mobile charge carriers [43]. The ionic conductivity σ_NMR_ increased from 0.55 mS/cm for *x* = 0.0 to 2.56 mS/cm for Li_6.5_As_0.5_Ge_0.5_S_5_Br (*x* = 0.5). A comparison of the ionic conductivities obtained from EIS and NMR is provided in Table S7. The corresponding Haven ratios are in the range of approximately 0.18–0.27, indicating correlated Li‐ion transport in the Li_6+x_As_1−x_Ge_x_S_5_Br series. These values are comparable to those reported in a recent study on Li_6−x_PS_5−x_Br_1+x_, where the Haven ratio decreased slightly with increasing substitution and reached approximately [43] 0.23 at *x* = 0.5. Figure 3e illustrates the temperature‐dependent lithium diffusion coefficients for all samples, following an Arrhenius trend that allows us to determine the activation energy (E_A_), which varies from 0.32 eV (*x* = 0.0) to 0.282 eV (*x* = 0.5). The similarity in activation energies derived from EIS and PFG NMR reflects the migration energy landscape at the particle length scale, including the effect of grain boundaries. Figure 3f illustrates how expanding the lithium cages goes along with improved ionic conductivity, suggesting these are correlated. Overall, the expansion of the lattice parameters and the increase in tetrahedral volume enlarge the lithium cages (R_mean_), enhancing the connectivity between these cages and thereby improving ionic transport.

### 2.2 Molecular Dynamics Simulations

To further investigate the Ge substitution, we performed an ab initio DFT‐based molecular dynamics (AIMD) simulation on calculations on representative supercell structures featuring the same S/Br disorder with four distinct As/Ge substitution rates (0, 25%, 50%, and 100% Ge content). Although the fully Ge‐substituted system (100%) is not experimentally accessible, evaluating this structure can provide valuable insight into the underlying diffusion mechanisms. AIMD simulations were performed starting from the geometry‐optimized supercells at 650 K for a simulation duration of 150 ps, providing information on the average Li distribution, site occupation, and diffusion mechanisms.

The obtained Li occupations and cage radii closely match experimental observations, confirming the structural picture discussed above based on neutron diffraction. As shown in Figure S14, the Li occupancy at the T4 site increases with increasing Ge content. Correspondingly, the average radii of the 4d Li‐ion cage increase, while the 4a Li‐ion cage radii remain constant (Figure S14). The RDF analysis indicates the same distances between S/Br at the 4d site and lithium with increasing Ge content, suggesting that the enlargement of the average 4d cage radius originates from increased lithium occupancy at the T4 position. Analysis of lithium MSDs reveals that diffusivity is lowest in the fully As‐substituted, while increasing with Ge substitution (Figure S14). Notably, diffusivity saturates at 50% Ge substitution, as evidenced by identical MSD values for 50% and 100% Ge‐substituted structures, indicating that additional increases in Ge content would not significantly enhance lithium diffusivity.

**FIGURE 4:**
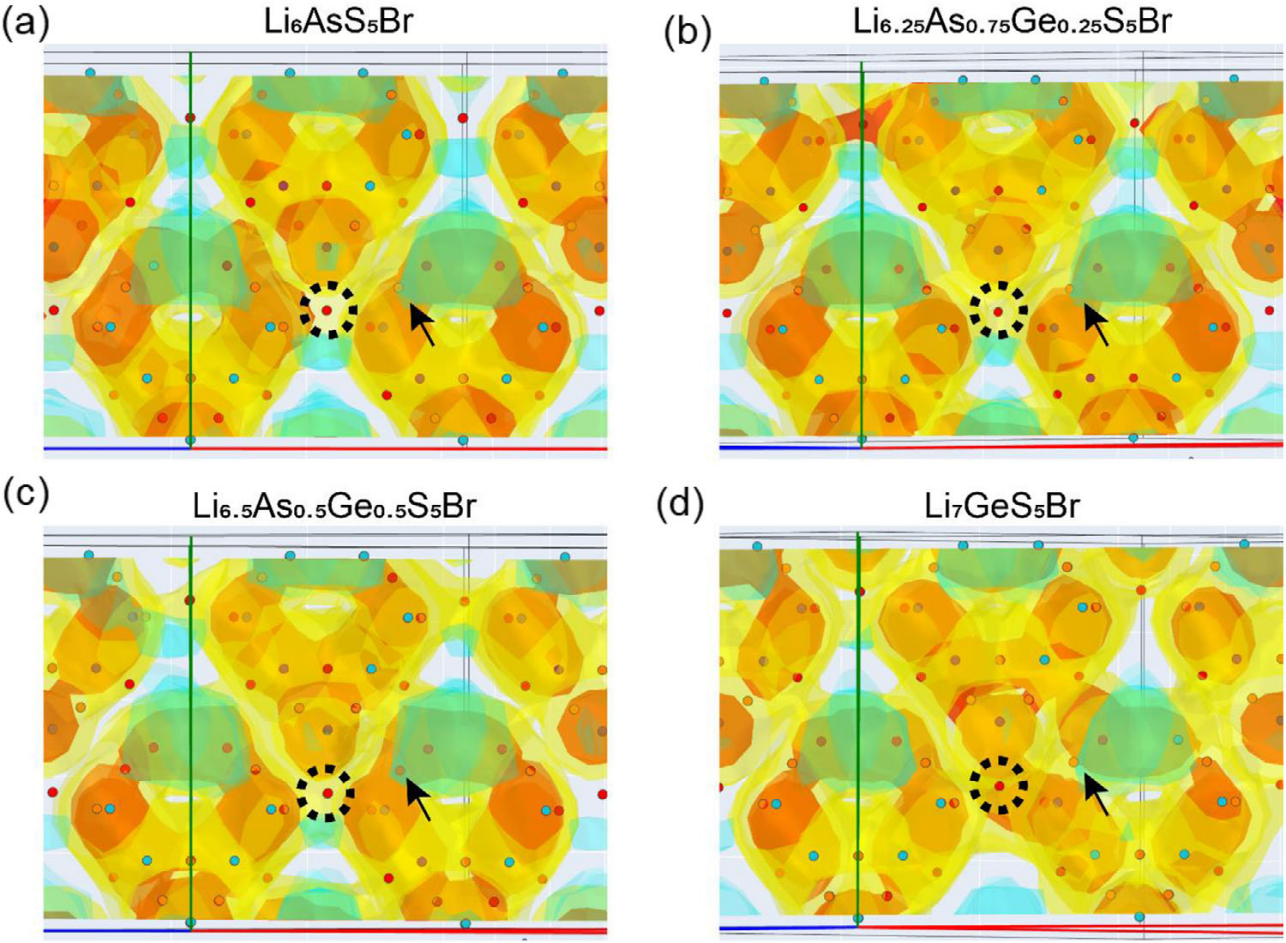
Probability density of Li^+^ obtained from AIMD simulations performed at 650 K. The area encircled by the black dashed line indicates an increase in lithium density between adjacent cages, while the black arrow highlights a decrease in lithium density for the intracage transport. The structures presented are: (a) Li_6_AsS_5_Br; (b) Li_6.25_As_0.75_Ge_0.25_S_5_Br; (c) Li_6.5_As_0.5_Ge_0.5_S_5_Br; (d) Li_7_GeS_5_Br.

Li transport was further qualitatively examined using Li density plots from AIMD simulations (Figure 4). In the fully As‐substituted system (0% Ge), lithium forms well‐defined cages around the 4d positions but lacks intercage connections, limiting macroscopic diffusion. With increasing Ge substitution, intercage connections emerge, facilitating Li transport. Interestingly, intracage jumps decrease, suggesting partial disconnection within a single cage; however, this effect does not hinder overall macroscopic lithium diffusion.

To understand this behavior, we performed detailed analyses of site‐specific lithium jumps within the lattice. This approach enables assessment of percolation pathways under different substitution conditions by dissecting Li transport into discrete jump environments. Such analysis recently provided valuable insights into disordered electrolytes, such as Li_2+x_S_1‐x_N_x_ and Li_6_PS_5_Br systems [58, 59], emphasizing the significant impact of local anionic environments on lithium percolation.

**FIGURE 5:**
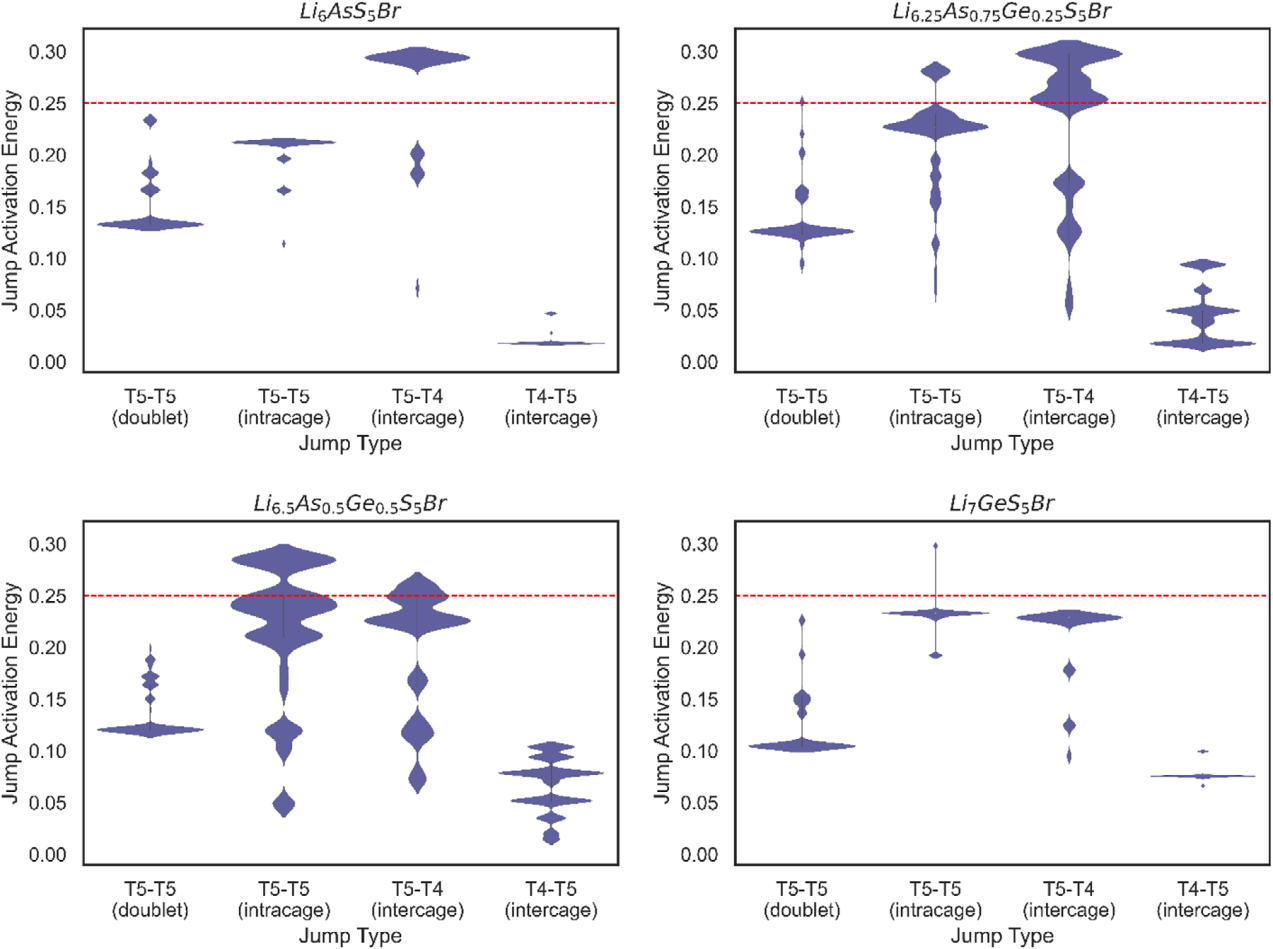
Comparison of jump activation energies per possible jump environment within specified composition, calculated from AIMD at 650 K. The average jump activation energy, calculated across three 2 × 1 × 1 supercells with different S/Br and As/Ge distribution, is shown in blue as “violins”. The width of the violins represents the relative occurrence of each environment. The structures presented are: (a) Li_6_AsS_5_Br; (b) Li_6.25_As_0.75_Ge_0.25_S_5_Br; (c) Li_6.5_As_0.5_Ge_0.5_S_5_Br; (d) Li_7_GeS_5_Br.

Disorder created by S/Br and As/Ge distribution in the argyrodite structure significantly affects lithium transport by introducing variations in the local environment that lithium encounters during a single jump. To simplify the analysis, we focused only on the jumps through the T5 and T4 sites, as intercage T5–T4–T5 jumps generally exhibit lower activation energies compared to T2–T2 jumps. Local environments were characterized based on the coordination of key lithium sites (T5 and T4) by AsS4^3^^−^/GeS4^4^^−^ tetrahedra and Br^−^/S^2^^−^ anions. The T5 tetrahedra are formed by two S^2−^ ions (corner‐shared with AsS_4_^3–^/GeS_4_^4–^ tetrahedra) and two Br–/S^2–^ anions at the 4a and 4d sites. T4 sites are coordinated by three S^2−^ ions (also corner‐shared with AsS_4_^3–^/GeS_4_^4–^ tetrahedra) and one Br–/S^2–^ anion at the 4a site, suggesting a larger influence of Ge substitution on the T4 position compared to that on the T5. For the environment‐specific analysis, four elements, according to the discussed coordination, were identified for each lithium position T5 (two cations at 4b, one anion at 4d, and one anion at 4a) and T4 (three cations at 4b, one anion at 4a). Violin plots (Figure 5) illustrate the distribution in jump activation energies, where the violin width reflects the occurrence frequency of each environment.

For Li_6_AsS_5_Br and Li_7_GeS_5_Br, local environments represent S/Br distributions, as 4b sites are occupied exclusively by As or Ge. Similar to Li_6_PS_5_Br^59^ at lower S/Br disorder rates, Li_6_AsS_5_Br exhibits rate‐limiting T5–T4 intercage jumps, consistent with Li density plots indicating limited long‐range diffusion and restricted accessibility to the T4 site. Substitution of P^5+^ by As^5+^ reduces average activation energies, enabling percolation at lower energy thresholds. For Li_6_AsS_5_Br, all jump types become accessible at approximately 0.3 eV, closely matching experimentally observed values (0.32 eV from PFG NMR).

Partial substitution of As^5+^ by Ge^4+^ creates a diverse range of environments where both intra‐ and intercage jumps become increasingly possible. By selecting an indicative energy threshold of approximately 0.25 eV, we observe that in the 25% Ge substitution case, percolation slightly improves, although most intercage jumps still exceed the threshold, aligning with modest MSD improvements. By 50% Ge substitution, most jump types fall below the threshold of 0.25 eV, enabling percolation. Notably, T5–T5 intracage jump seems to be rate‐limiting, explaining partly‐disconnected cages visible at the lithium density analysis. All T5–T5 doublet and T5–T4 intercage jump environments become accessible at approximately 0.27 eV, aligning with NMR results of 0.282 eV and suggesting that T5–T5 intracage jump does not slow down the percolation. At the experimentally inaccessible 100% Ge‐substituted structure, fewer distinct environments exist, but all site‐specific jumps fall below 0.25 eV, indicating a decreased average activation energy for P^5+^ to As^5+^ and As^5+^ to Ge^4+^ substitutions.

To better understand the interactions inside the structure, we extracted Pauling electronegativity values, electronegativity decreases in the following order: P^5+^ (χ = 2.19), As^5+^ (χ = 2.18), Ge^4+^ (χ = 2.01), which implies that Ge forms more polar covalent bonds with sulfur in GeS_4_^4–^ compared to PS_4_^3–^. This increases sulfur's partial negative charge in GeS_4_^4–^ tetrahedra, suggesting stronger Coulombic interaction towards the Li^+^ ions. This stronger sulfur–lithium interaction counterbalances the Li^+^ attraction to S^2^^−^ sites at 4d, preventing lithium from becoming trapped within individual cages. In addition to the correlated shorter intercage jump distances, this change in atomic scale interactions is held responsible for improving macroscopic lithium mobility, consistent with experimental observations and computational results.

### 2.3 Air Stability

**FIGURE 6:**
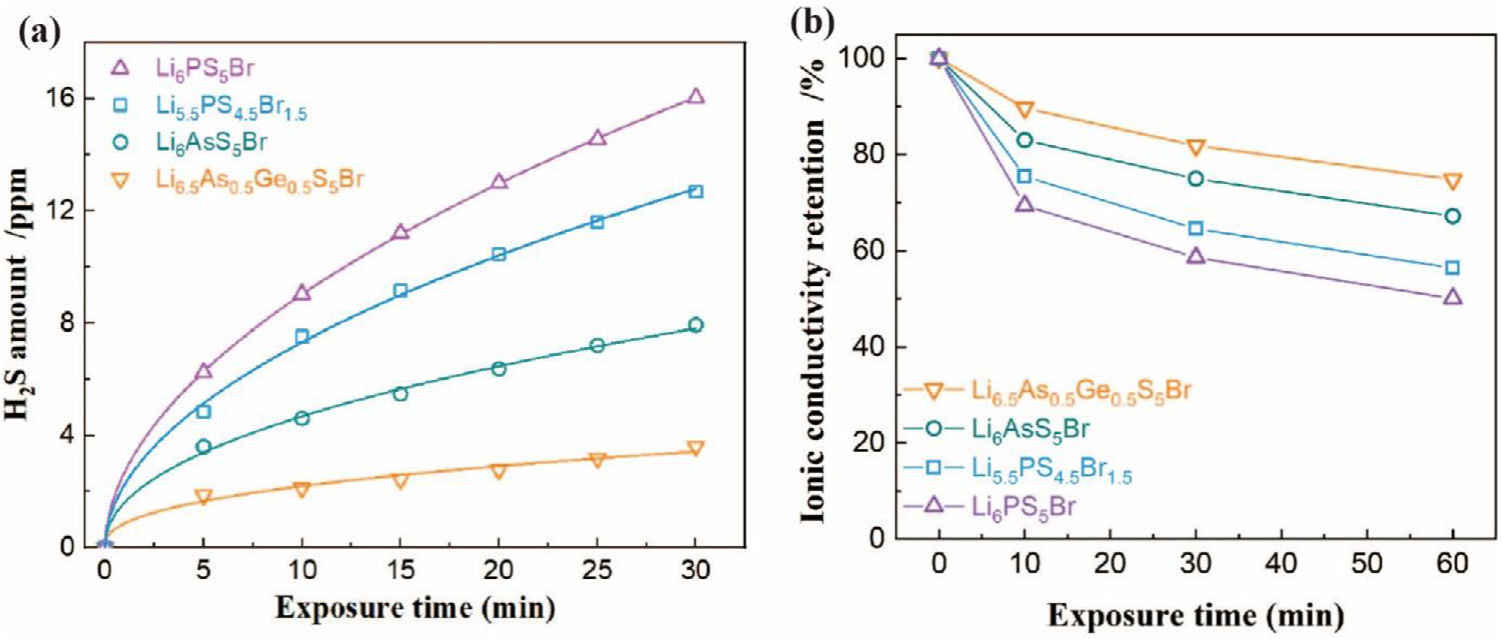
(a) The quantity of H_2_S released from Li_6.5_As_0.5_Ge_0.5_S_5_Br, Li_6_AsS_5_Br_,_ Li_5.5_PS_4.5_Br_1.5_, and Li_6_PS_5_Br solid electrolytes as a function of exposure time in 40% nitrogen‐humidified environment at room temperature. (b) Ionic conductivity retention of the above sulfides after exposure to moist air with a humidity of 20% at room temperature for different periods of time.

One of the most significant challenges to the extensive practical application of sulfide solid electrolytes is their susceptibility to deterioration in a humid atmosphere. Exposure to moisture causes sulfide solid electrolytes to release H_2_S gas, resulting in structural deterioration and capacity degradation as well as safety hazards. As illustrated in Figure 6a, the Li_6_PS_5_Br electrolyte exhibits the highest H_2_S release amount when subjected to a 40% humidity nitrogen environment for 30 min, exhibiting the fastest release rate. Increasing bromine content reduces both the amount of released H_2_S and the release rate. This is likely due to a decrease of free S^2−^ (S^2−^ not bound to P^5+^) ions in the argyrodite crystal structure caused by excessive Br substitution. Substituting P with As also reduces H_2_S release. Additionally, substituting As with Ge in the Li_6_AsS_5_Br will further decrease the H_2_S release rate. It is noteworthy that the Li_6.5_As_0.5_Ge_0.5_S_5_Br electrolyte shows the lowest H_2_S release, which is only a quarter of the amount released by the Li_6_PS_5_Br electrolyte.

Given the greater practical importance of ionic conductivity in sulfide electrolytes, the Li_6.5_As_0.5_Ge_0.5_S_5_Br, Li_6_AsS_5_Br, Li_5.5_PS_4.5_Br_1.5_, and Li_6_PS_5_Br electrolytes were subjected to an atmospheric environment with a relative humidity of 20% to evaluate their stability in air. As illustrated in Figure 6b, during the exposure period, the ionic conductivity initially declines rapidly before slowing down. It is observed that the ionic conductivity of the Li_6_PS_5_Br electrolyte decreases rapidly during the initial 10 min, indicating a more pronounced reaction with moisture. After one hour of exposure, the ionic conductivity retention follows the order: Li_6.5_As_0.5_Ge_0.5_S_5_Br > Li_6_AsS_5_Br > Li_5.5_PS_4.5_Br_1.5_> Li_6_PS_5_Br. The Li_6.5_As_0.5_Ge_0.5_S_5_Br electrolyte shows obvious enhanced air stability. The Raman spectra of the sulfide electrolytes before and after exposure to the air remain unchanged, indicating stability. All samples consistently exhibited peaks assigned to PS_4_^3−^ and GeS_4_^4−^ and AsS_4_^3−^, as illustrated in Figure S15. A partial amorphous phase might form on the surface of sulfide particles, leading to a decrease in the ionic conductivity of sulfide electrolytes after exposure to humid air. To the best of our knowledge, Li_6.5_As_0.5_Ge_0.5_S_5_Br is one of the few reported sulfide electrolytes that demonstrates both exceptional air stability and high room‐temperature ionic conductivity.

### 2.4 Battery Performance

**FIGURE 7:**
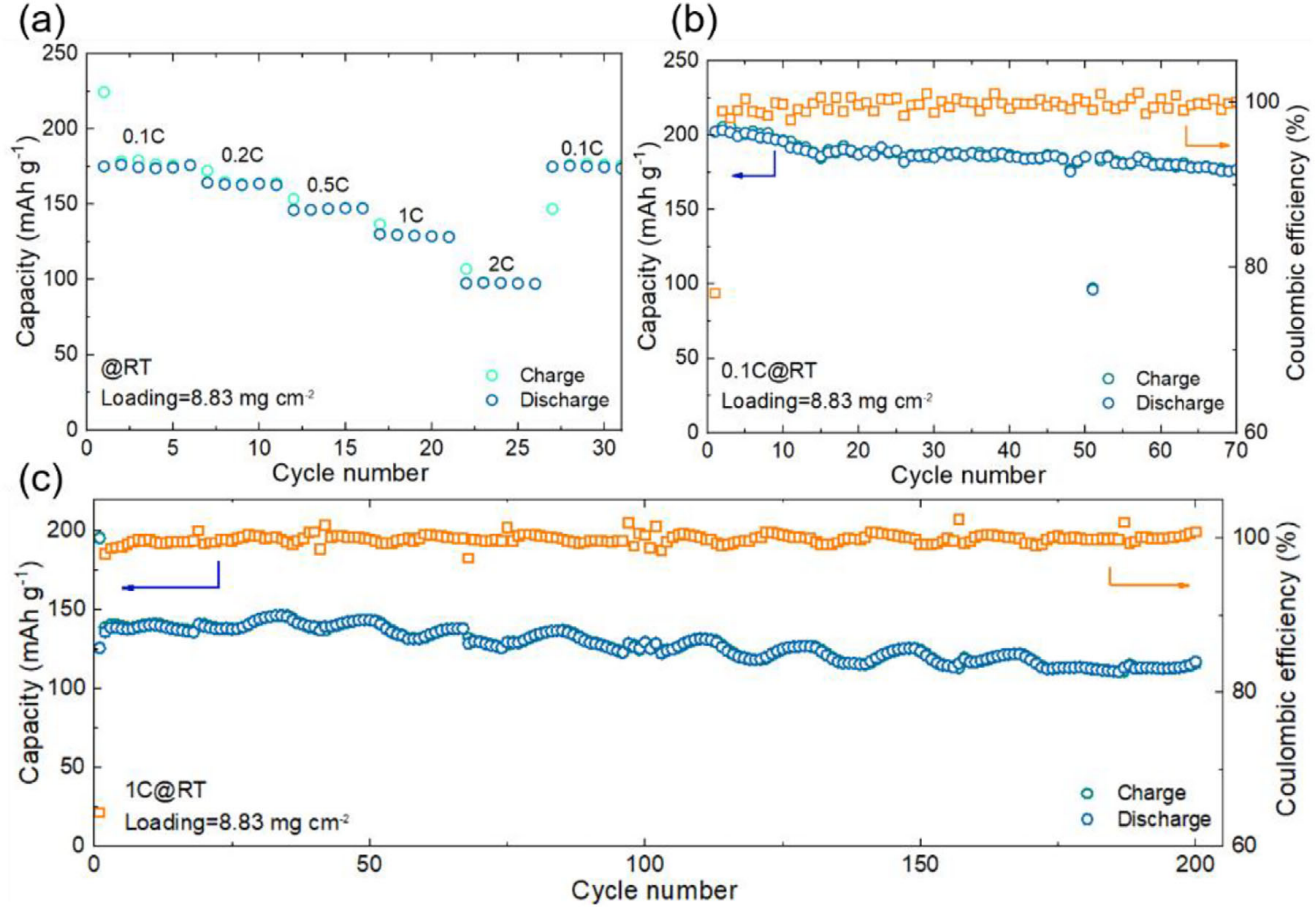
(a) Rate performance of an In/InLi| Li_6.5_As_0.5_Ge_0.5_S_5_Br |s‐NCM83‐Li_6.5_As_0.5_Ge_0.5_S_5_Br‐C65 cell, Cycling performance of an In/InLi| Li_6.5_As_0.5_Ge_0.5_S_5_Br |s‐NCM90‐Li_6.5_As_0.5_Ge_0.5_S_5_Br‐C65 cell at (b) 0.1C and (c) 1C, respectively.

To demonstrate the application of Li_6.5_As_0.5_Ge_0.5_S_5_Br in ASSBs, the cells with LiNi_0.9_Co_0.06_Mn_0.04_O_2_@Li_3_BO_3_ (NCM90@Li_3_BO_3_) or LiNi_0.83_Co_0.11_Mn_0.06_O_2_@Li_3_BO_3_ (NCM83@Li_3_BO_3_) as cathode active materials and In/InLi as anode were assembled. Figure 7a shows the rate performance of In/InLi| Li_6.5_As_0.5_Ge_0.5_S_5_Br | NCM83@Li_3_BO_3_‐C65‐ Li_6.5_As_0.5_Ge_0.5_S_5_Br cell with cathode active material loading of 8.83 mg cm^−2^ at room temperature (RT). The cell delivers discharge capacities of 174.4, 163.7, 147.4, 128.7, and 97.4 mAh g^−1^ at 0.1C, 0.2C, 0.5C, 1C and 2C, respectively. Subsequent cycling at 0.1C, recovers a reversible capacity of 174.7 mAh g^−1^, corresponding to a capacity retention of 100.2% related to the initial cycle, thus demonstrating excellent reversibility. Figure 7b shows the cycling performance of the In/InLi| Li_6.5_As_0.5_Ge_0.5_S_5_Br | NCM90@Li_3_BO_3_‐C65‐ Li_6.5_As_0.5_Ge_0.5_S_5_Br cell. The cell delivers an initial charge and discharge capacity of 263.3 and 202.4 mAh g^−1^ at 0.1C at RT. After 70 cycles, the discharge capacity degrades to 176.7 mAh g^−1^, corresponding to a high capacity retention of 87.3%. The voltage profiles of the cells at different cycles (0.1C) are shown in Figure S16a. A slight overpotential increases after 70 cycles. To showcase the cycling performance of the ASSBs at high current densities, the NCM90 cell was cycled at 1C at RT (Figure 7c). A high specific capacity of 117 mAh g^−1^ was achieved after 200 cycles without any obvious capacity degradation. Further increasing the C‐rate to 2C, the cell also delivers a stable discharge capacity of 100.4 mAh g^−1^ after 80 cycles (Figure S16c). Good overlap between the 2nd and 80th charge/discharge curves further demonstrates high capacity retention at large current densities (Figure S16d). The above results demonstrate that Li_6.5_As_0.5_Ge_0.5_S_5_Br is a promising solid electrolyte candidate for ASSBs application. When evaluating the toxicity and environmental impact of arsenic‐containing ion conductors such as Li_6.5_As_0.5_Ge_0.5_S_5_Br and Li_6_AsS_5_Br, both historical context and chemical properties should be considered. Arsenic is a toxic metalloid that occurs widely in the environment in various oxidation states and chemical forms, including inorganic salts, organic compounds, and gaseous species [50, 51]. The maximum permissible level of arsenic in drinking water is 10 ppb, as established by the World Health Organization and the US EPA [60]. Although arsenic oxides are highly toxic, arsenic sulfides, specifically realgar (As_4_S_4_) and As_2_S_3_, are considered significantly less toxic than inorganic arsenic oxides [61, 62]. However, amorphous arsenic sulfides may exhibit higher environmental toxicity, and transformation into more soluble and toxic forms under certain conditions remains possible [61, 63, 64]. In addition, despite the high toxicity of arsine gas, it is still used in the production of gallium arsenide, an important material for semiconductors, lasers, light‐emitting diodes, and microwave devices [64]. Therefore, Safety protocols for handling and disposal are necessary, and comprehensive toxicity and environmental studies are essential for the safe and sustainable use of these materials.

## 3 Conclusion

In conclusion, we successfully synthesized, analyzed, and characterized the Ge‐substituted thioarsenate argyrodite series, Li_6+x_Ge_x_As_1−x_S_5_Br. Structural insights were gained using x‐ray and neutron diffraction, analyzed by Rietveld refinements, while the transport properties and mechanism were assessed through temperature‐dependent impedance spectroscopy, ^7^Li PGF NMR, and ab initio molecular dynamics (AIMD) simulations. The incorporation of the larger Ge^4+^ cation led to notable increases in both polyhedral‐ and unit cell volumes, alongside significant changes in lithium distribution, up to a solubility limit of *x* = 0.5. The addition of Ge^4+^ strongly improves the lithium ionic conductivity of Li_6+x_Ge_x_As_1−x_S_5_Br, achieving a 6‐fold increase to a maximum of 12.7 mScm^−1^ at *x* = 0.5 compared to the conductivity of the Li_6_AsS_5_Br end member. Increasing the Ge substitution leads to higher Li occupancy at T4 sites, expanded 4d cage radii, and enhanced intercage lithium diffusion. Notably, lithium diffusivity significantly improves up to *x* = 0.5 Ge substitution, beyond which no further increase is observed, in line with the AMD simulations. Detailed analysis of site‐specific lithium jumps revealed that Ge substitution lowers the activation energies, promoting intercage lithium transport and overall percolation. This enhancement is attributed to the increased polarity of Ge─S bonds compared to As─S bonds, reducing lithium trapping within cages and improving macroscopic lithium diffusion. In addition, Li_6.5_As_0.5_Ge_0.5_S_5_Br exhibits better air stability compared with Li_6_PS_5_Br, Li_5.5_PS_4.5_Br_1.5_, Li_6_AsS_5_Br, thus combining high conductivity with substantially improved air stability Hereby, this work has revealed the relationship between the lithium sub‐structure and the ionic transport mechanism of lithium thioarsante argyrodite electrolyte, paving the way for the development of innovative sulfide solid electrolytes that exhibit both enhanced ionic conductivity and air stability.

## Conflicts of Interest

The authors declare no conflicts of interest.

## Supporting information




**Supporting File**: smll74170‐sup‐0001‐SuppMat.docx.

## Data Availability

The data that support the findings of this study are available in the supplementary material of this article.
