# Supplementary material for: Superionic Germanium Substituted Lithium Thioarsante Li6+ x Ge x As1‐ x S5Br Argyrodites With High Air Stability
Source: Small. 2026 Jul 30;22(45):e74170. doi: 10.1002/smll.74170 (PMC13472247; doi:10.1002/smll.74170)
Supplement: Supplementary file 1 [file SMLL-22-e74170-s001.docx]

**Superionic Germanium Substituted Lithium thioarsante Li_6+_*_x_*Ge*_x_*As_1-_*_x_*S_5_Br Argyrodites with High Air Stability**

Ajay Gautam*^a,c, #,^*^*^*^,^*, Ruihua Zhou*^b^*^,^ *^#,^*, Anastasia K. Lavrinenko*^a^*, Hanan Al-Kutubi*^a^*, Swapna Ganapathy*^a^*, Xin Zhang*^b^*, Alexandros Vasileiadis*^a^*, Shuo Wang*^b^*^, *^, Marnix Wagemaker*^a,^* ^*^

*^a^Storage of Electrochemical Energy, Department of Radiation Science and Technology, Faculty of Applied Sciences, Delft University of Technology, Mekelweg 15, 2929JB, Delft, The Netherlands*

*^b^State Key Laboratory of Advanced Glass Materials, School of Material Science and Engineering, Wuhan University of Technology, Wuhan, 430070 China*

*^c^Materials Engineering, Indian Institute of Technology Jammu, NH-44, PO Nagrota, Jagti, Jammu and Kashmir 181221 India*

^#^These authors contributed equally.

*Corresponding authors: [m.wagemaker@tudelft.nl](mailto:m.wagemaker@tudelft.nl),

[shuowang@whut.edu.cn](mailto:shuowang@whut.edu.cn)

[Ajay.gautam@iitjammu.ac.in](mailto:Ajay.gautam@iitjammu.ac.in)

**Computational Details**

Density functional theory (DFT) calculations based on the Perdew–Burke–Ernzerhof functional for solid-state systems (PBEsol)^1,2^ within the Vienna Ab initio Software Package (VASP 6.3.2)^3^ were utilized. Projector augmented wave (PAW)^4^ potentials were used with cores of [He] for Li, [Ne] for P and S, and [Ar] for Br. Structure optimizations were conducted with an energy cutoff of 340 eV in 2x1x1 argyrodite supercells.

Four argyrodite structures Li_6_AsS_5_Br, Li_6.25_As_0.75_Ge_0.25_S_5_Br, Li_6.5_As_0.5_Ge_0.5_S_5_Br, and Li_7_GeS_5_Br were generated with 12.5% of S/Br disorder. Since multiple orderings of S/Br and As/Ge in the sublattices are possible, several unique configurations with random distributions were optimized for all structures.

Ab initio molecular dynamics (AIMD) simulations in the canonical (NVT) ensemble using the Nosé-Hoover thermostat^5,6^ were performed at 900 K. The energy cutoff was reduced to 300 eV, and gamma-only k-point mesh was used. The selected time step was 2 fs for a total computational time of 150 ps. Site-sensitive properties such as site-specific jump frequencies, and energy barriers were obtained using the analysis tools developed in our group^7,8^. To analyze the individual jumps, we defined two types of Li positions in the crystal lattice, namely, Wyckoff 48h and Wyckoff 16e^9^, counting the times Li resides in these positions throughout the simulation as well as the number of jumps between these positions. To analyze Li-ion jump activation energy specific to the local environments S/Br and As/Ge, we examined AIMD simulations at 650 K for three selected supercells for each composition.

**Experimental:**

**Sample Preparation.** The Li_6+x_As_1-x_Ge_x_Br (for 0.0 ≤ x ≤0.6) solid electrolytes were prepared by solid-state reaction. The Li_2_S (Wuhan Tianshi Kefeng New Energy Technology Co., Ltd., 99.9%), As_2_S_3_ (Innochem, 99.9%), GeS_2_ (Innochem, 99.99%), Sulfur (Aladdin, 99.99%), and LiBr (Acros, 99%) as the starting materials were weighed in an appropriate ratio, and then mixed using a planetary ball mill (FRITSCH PULVERISETTE 7 premium line) at 100 rpm for 2 h. Afterward, the mixture was pressed into pellets and sealed in quartz ampoules using a vacuum sealing machine (VS-Q2, Guangzhou SunJune technology Co. Ltd.). Next, the ampoules were annealed at 550 °C for 6 h and then cooled down to room temperature naturally. Finally, the pellets were manually ground to the fine powder.

**X-ray Diffraction (XRD):** The phase and purity of the lithium argyrodite samples were analyzed using a SmartLab SE diffractometer (Rigaku Corporation) equipped with a Cu Kα radiation source. Data collection was performed across a 2θ range of 10° to 90°. To prevent exposure to ambient air and moisture, the samples were mounted in an airtight sample holder sealed with Kapton polyimide film inside an argon-filled glovebox.

**Neutron Powder Diffraction (NPD):** Room temperature neutron diffraction experiments were conducted on the PEARL diffractometer at the TU Delft research reactor, employing the 533 reflection from a germanium monochromator, yielding a neutron wavelength of λ = 1.667 Å. Approximately 1.5 g of each sample was loaded into a 6-mm diameter cylindrical vanadium canister under an argon atmosphere and sealed with indium wire to maintain an inert environment during transfer. Measurements were carried out for 6–18 hours under vacuum, covering a 2θ range from 10° to 160°. Data from an empty vanadium can were used as background and subtracted from each diffraction profile.

**Rietveld Refinement:** Rietveld analysis of the neutron diffraction patterns was carried out using the TOPAS V7 software. Structural models were initially based on literature data for Li₆PS₅Br (ref.^10^). Fit quality was assessed using the Rwp and goodness-of-fit (GOF) parameters. Background contributions were modeled with a 15-term Chebyshev polynomial, while peak shapes were described using a modified pseudo-Voigt function. Refined parameters included scale factor, zero-point correction, unit cell parameter, isotropic atomic displacement parameters, and site occupancies of Ge and As cation, with occupancy constraints to maintain charge neutrality. Lithium site occupancies were included, omitting those with negative thermal parameters (assumed below the detection limit). Final lithium content was adjusted in accordance with the refined Ge occupancy. Full details on refinement constraints are provided in the Supplementary Tables S2–S6.

**Scanning Electron Microscopy (SEM):** Particle morphology was characterized by SEM (JSM-7610F) operated at 15 kV. Elemental analysis was performed concurrently using energy-dispersive X-ray spectroscopy (EDS).

**Air Stability Measurements**: 100 mg of the Li_6.5_As_0.5_Ge_0.5_S_5_Br, Li_6_AsS_5_Br, Li_5.5_PS_4.5_Br_1.5_, and Li_6_PS_5_Br electrolyte powders were compressed at 150 MPa to form 10 mm pellets. The pellets were then exposed to a 40% relative humidity nitrogen environment for a duration of 30 minutes within a sealed container with a volume of 40 L. The quantity of H_2_S gas produced was determined through the measurement of its concentration using an H_2_S gas sensor (RA2000-H2S, Shenzhen Ruian Electronic Technology Co., Ltd). Furthermore, Raman and ionic conductivity measurements were conducted on all samples prior to and following exposure to humid air (20% relative humidity) to assess the air stability.

**All-solid-state batteries assembly**: The composite cathode was prepared by hand mixing single crystal LiNi_0.9_Co_0.06_Mn_0.04_O_2_ (s-NCM90@Li_3_BO_3_, provided by BASF Shanshan Battery Materials Co., Ltd.) or LiNi_0.83_Co_0.11_Mn_0.06_O_2_ (s-NCM83), Li_6.5_As_0.5_Ge_0.5_S_5_Br and Super C65 carbon black in a weight ratio of 70:30:1 for 1 h. Firstly, 80 mg of Li_6.5_As_0.5_Ge_0.5_S_5_Br powers were pressed into pellet to form the separator under 100 MPa. Subsequently, 10 mg of composite cathode was spread on one separator layer and pressed under 100 MPa. After that, an indium foil and a lithium foil were sequentially attached to the other side of the separator and pressed at 50 MPa. Finally, the battery was pressed at 150 MPa for 3 min. During cycling, a stack pressure of ~100 MPa was applied to the cells. Galvanostatic cycling of the cells was conducted in a potential range of 3–4.3 V *vs*. Li^+^/Li for the NCM90 and 3–4.25 V *vs*. Li^+^/Li for the NCM83 cathode active material at room temperature using a LANHE battery testing system (CT3001A) and where 1C = 200 mA g^−1^ was used for battery testing.

***Figure S1:*** *Lithium argyrodite structure Li_6_AsS_5_Br, where Free S^2−^ and Br^−^ ions are occupied the 4d and 4a site, The 132 interstitial voids can potentially be occupied by lithium, forming lithium tetrahedra Li(S/X)_4_, which connect (-face, edge, corner) with the PS_4_^3−^ tetrahedra, designated as T1-T5a^10,11^*

***Figure S2****: Rietveld refinement analysis of neutron diffraction (Left) and x-ray diffraction data (Right) for Li_6_AsS_5_Br. The experimental data is represented by black dots, the calculated pattern is shown as red lines, and the blue lines depict the difference profile between the experimental and calculated data. The orange tick marks indicate the expected peak positions of Li_6_AsS_5_Br.*

***Figure S3:*** *Rietveld refinement analysis of neutron diffraction and x-ray diffraction data for Li_6.2_As_0.8_Ge_0.2_S_5_Br composition. The experimental data is represented by black dots, the calculated pattern is shown as red lines, and the blue lines depict the difference profile between the experimental and calculated data. The orange tick marks indicate the expected peak positions of Li_6.2_As_0.8_Ge_0.2_S_5_Br.*

***Figure S4:*** *Rietveld refinement analysis of neutron diffraction and x-ray diffraction data for Li_6.3_As_0.7_Ge_0.3_S_5_Br composition. The experimental data is represented by black dots, the calculated pattern is shown as red lines, and the blue lines depict the difference profile between the experimental and calculated data. The orange tick marks indicate the expected peak positions of Li_6.3_As_0.7_Ge_0.3_S_5_Br.*

***Figure S5:*** *Rietveld refinement analysis of neutron diffraction and x-ray diffraction data for Li_6.4_As_0.6_Ge_0.4_S_5_Br composition. The experimental data is represented by black dots, the calculated pattern is shown as red lines, and the blue lines depict the difference profile between the experimental and calculated data. The orange, dark brown, and purple tick marks indicate the expected peak positions of Li_6.4_As_0.6_Ge_0.4_S_5_Br, LiBr, and Li_3+x_As_1-x_Ge_x_S_4_ respectively.*

***Figure S6:*** *Rietveld refinement analysis of x-ray diffraction data for Li_6.6_As_0.4_Ge_0.6_S_5_Br composition. The experimental data is represented by black dots, the calculated pattern is shown as red lines, and the blue lines depict the difference profile between the experimental and calculated data. The orange, dark brown, and purple tick marks indicate the expected peak positions of Li_6.6_As_0.4_Ge_0.6_S_5_Br, LiBr, and Li_3+x_As_1-x_Ge_x_S_4_ respectively.*

***Figure S7.*** *SEM images (a-d) of the Li_6.5_As_0.5_Ge_0.5_S_5_Br, Li_6_AsS_5_Br, Li_5.5_PS_4.5_Br_1.5_, Li_6_PS_5_Br solid electrolytes and corresponding germanium (e), arsenic (f, g), phosphorus (h, i), sulfur (j-m) and bromine (n-q) EDX mappings.*

***Figure S8.*** *Lithium argyrodite Li_6_As_5_Br framework, two cages are displayed to demonstrate the connectivity within the structure: (a) shows the connections occurring via T5 sites through the T4 site, and (b) highlights the T2-T2 distance diffusion pathways, elucidating potential pathways for lithium ion diffusion.*

***Figure S9.*** *Illustrates the lithium site occupancy across the Ge substitution in the thioarsenate argyrodite Li_6+x_GeₓAs₁₋ₓS₅Br series.*

***Figure S10.*** *Lithium tetrahedral volume of different Li sites present in the Ge substitution in the thioarsenate argyrodite Li_6+x_GeₓAs₁₋ₓS₅Br series.*

***Figure S11.*** *Lithium jump pathways in the thioarsenate argyrodite Li_6+x_GeₓAs₁₋ₓS₅Br series, showing a pathway connecting two T5 sites via a T4 site (T5−T4−T5), and a second pathway through two neighboring T2 sites (T5−T2−T2−T5) present due to Ge substitution.*

| Atom | Wyckoff site | *x/a* | y/*b* | z/*c* | Occ | B_eq_/ Å^2^ |
| --- | --- | --- | --- | --- | --- | --- |
| LiT5 | 48*h* | *Pos1* | *Pos2* | *1-Pos1* | *Occ3* | *Var1* |
| LiT2 | 48*h* | *Pos3* | *Pos4* | *0.5+Pos4* | *((6+(1-Occ2)*4-48*Occ3-16*Occ5)/48* | *Var1* |
| LiT4 | 16*e* | *Pos6* | *Pos6* | *Pos6* | *Occ5* | *Var1* |
| As1 | 4*b* | 0 | 0 | 0.5 | 1 | *Var2* |
| Ge1 | 4*b* | 0 | 0 | 0.5 | 1-*Occ2* | *Var2* |
| S2 | 16*e* | *Pos7* | *-Pos7* | *0.5+Pos7* | *1* | *Var3* |
| Br1 | 4*a* | 0 | 0 | 1 | *Occ1* | *Var4* |
| Br2 | 4*d* | *0.25* | *0.25* | *0.75* | *1-Occ1* | *Var5* |
| S1 | 4*d* | 0.25 | 0.25 | 0.75 | *Occ1* | *Var5* |
| S3 | 4*a* | 0 | 0 | 1 | *1-Occ1* | *Var4* |

***Table S1.*** *Constraints used to refine Li_6+x_As_1-x_Ge_x_S_5_Br neutron diffraction data.*

***Table S2.*** *Crystallographic data of Li_6_AsS_5_Br sample. The lattice parameter, atomic positions, and occupancies are obtained from the Rietveld refinements against the neutron diffraction data. The uncertainty/error value is shown in brackets.*

| *a* = 10.1049(6) Å; F-43m  R_wp_ = 3.97 % ; λ = 1.667Å | | | | | | | |
| --- | --- | --- | --- | --- | --- | --- | --- |
| Atom | Position | x | y | z | Occ. | B/Å2 | *Wyckoff site* |
| Br | Br1 | 0.25 | 0.25 | 0.75 | 0.12(2) | 1.77(8) | 4*d* |
| Br | Br2 | 0 | 0 | 1 | 0.88(2) | 3.39(7) | 4*a* |
| S | S1 | 0 | 0 | 1 | 0.12(2) | 3.39(7) | 4*a* |
| S | S2 | 0.25 | 0.25 | 0.75 | 0.88(2) | 1.77(8) | 4*d* |
| S | S3 | 0.1230(4) | -0.1230(4) | 0.6230(4) | 1 | 2.16(9) | 16*e* |
| As | As1 | 0 | 0 | 0.5 | 1 | 1.27(5) | 4*b* |
| Ge | Ge1 | - | - | - | - | - |  |
| Li | T5 | 0.3055(8) | 0.0260(2) | 0.6945(8) | 0.465(5) | 3.4(2) | 48*h* |
| Li | T2 | 0.295(6) | 0.087(6) | 0.587(6) | 0.05671(8) | 3.4(2) | 48*h* |
| Li | T4 | 0.151(2) | 0.151(2) | 0.151(2) | 0.020(4) | 3.4(2) | 16*e* |

***Table S3.*** *Crystallographic data of Li_6.2_As_0.8_Ge_0.2_S_5_Br sample. The lattice parameter, atomic positions, and occupancies are obtained from the Rietveld refinements against the neutron diffraction data. The uncertainty/error value is shown in brackets.*

| *a* = 10.1301(3) Å; F-43m  R_wp_ =5.15 % ; λ = 1.667Å | | | | | | | |
| --- | --- | --- | --- | --- | --- | --- | --- |
| Atom | Position | x | y | z | Occ. | B/Å2 | *Wyckoff site* |
| Br | Br1 | 0.25 | 0.25 | 0.75 | 0.11(1) | 1.59(9) | 4*d* |
| Br | Br2 | 0 | 0 | 1 | 0.89(2) | 3.66(2) | 4*a* |
| S | S1 | 0 | 0 | 1 | 0.11(1) | 3.66(2) | 4*a* |
| S | S2 | 0.25 | 0.25 | 0.75 | 0.89(2) | 1.59(9) | 4*d* |
| S | S3 | 0.1231(4) | -1231(4) | 0.6231(4) | 1 | 1.66(9) | 16*e* |
| As | As1 | 0 | 0 | 0.5 | 0.8 | 1.28(6) | 4*b* |
| Ge | Ge1 | 0 | 0 | 0.5 | 0.2 | 1.28(6) | 4*b* |
| Li | T5 | 0.3085(9) | 0.0263(9) | 0.6915(9) | 0.469(9) | 3.1(2) | 48*h* |
| Li | T2 | 0.290(9) | 0.440(8) | 0.940(8) | 0.627(6) | 3.1(2) | 48*h* |
| Li | T4 | 0.150(1) | 0.150(1) | 0.150(1) | 0.045(9) | 3.1(2) | 16*e* |

***Table S4.*** *Crystallographic data of Li_6.3_As_0.7_Ge_0.3_S_5_Br sample. The lattice parameter, atomic positions, and occupancies are obtained from the Rietveld refinements against the neutron diffraction data. The uncertainty/error value is shown in brackets.*

| *a* = 10.13850(9) Å; F-43m  R_wp_ = 3.69 % ; λ = 1.667Å | | | | | | | |
| --- | --- | --- | --- | --- | --- | --- | --- |
| Atom | Position | x | y | z | Occ. | B/Å2 | *Wyckoff site* |
| Br | Br1 | 0.25 | 0.25 | 0.75 | 0.14(2) | 1.41(9) | 4*d* |
| Br | Br2 | 0 | 0 | 1 | 0.86(2) | 3.85(9) | 4*a* |
| S | S1 | 0 | 0 | 1 | 0.14(2) | 3.85(9) | 4*a* |
| S | S2 | 0.25 | 0.25 | 0.75 | 0.86(2) | 1.41(9) | 4*d* |
| S | S3 | 0.1232(4) | -0.1232(4) | 0.6232(4) | 1 | 1.93(8) | 16*e* |
| As | As1 | 0 | 0 | 0.5 | 0.7 | 1.86(5) | 4*b* |
| Ge | Ge1 | 0 | 0 | 0.5 | 0.3 | 1.86(5) | 4*b* |
| Li | T5 | 0.312(1) | 0.0285(1) | 0.688(1) | 0.455(9) | 4.2(3) | 48*h* |
| Li | T2 | 0.249(6) | 0.4438(3) | 0.9438(3) | 0.106(8) | 4.2(3) | 48*h* |
| Li | T4 | 0.153(2) | 0.153(2) | 0.153(2) | 0.041(9) | 4.2(3) | 16*e* |

***Table S5.*** *Crystallographic data of Li_6.4_As_0.6_Ge_0.4_S_5_Br sample. The lattice parameter, atomic positions, and occupancies are obtained from the Rietveld refinements against the neutron diffraction data. The uncertainty/error value is shown in brackets.*

| *a* = 10.15211(9) Å; F-43m  R_wp_ = 3.30 % ; λ = 1.667Å  0.5 wt.% LiBr & 2.3 wt.% Li_4-x_Ge_1-x_As_x_S_4_ | | | | | | | |
| --- | --- | --- | --- | --- | --- | --- | --- |
| Atom | Position | x | y | z | Occ. | B/Å2 | *Wyckoff site* |
| Br | Br1 | 0.25 | 0.25 | 0.75 | 0.125(2) | 1.6(1) | 4*d* |
| Br | Br2 | 0 | 0 | 1 | 0.875(2) | 1.6(1) | 4*a* |
| S | S1 | 0 | 0 | 1 | 0.125(2) | 3.9(1) | 4*a* |
| S | S2 | 0.25 | 0.25 | 0.75 | 0.875(2) | 3.9(1) | 4*d* |
| S | S3 | 0.1237(8) | -0.1237(8) | 0.6237(8) | 1 | 1.68(9) | 16*e* |
| As | As1 | 0 | 0 | 0.5 | 0.6 | 1.31(5) | 4*b* |
| Ge | Ge1 | 0 | 0 | 0.5 | 0.4 | 1.31(5) | 4*b* |
| Li | T5 | 0.3058(9) | 0.0239(8) | 0.6942(9) | 0.479(9) | 3.2(2) | 48*h* |
| Li | T2 | 0.231(4) | 0.449(2) | 0.949(2) | 0.097(1) | 3.2(2) | 48*h* |
| Li | T4 | 0.146(4) | 0.146(4) | 0.146(4) | 0.06(2) | 3.2(2) | 16*e* |

***Table S6.*** *Crystallographic data of Li_6.5_As_0.5_Ge_0.5_S_5_Br sample. The lattice parameter, atomic positions, and occupancies are obtained from the Rietveld refinements against the neutron diffraction data. The uncertainty/error value is shown in brackets.*

| *a* = 10.15823(9) Å; F-43m  R_wp_ = 3.78 % ; λ = 1.667Å,  2 wt.% LiBr & 5 wt.% Li_4-x_Ge_1-x_As_x_S_4_ | | | | | | | |
| --- | --- | --- | --- | --- | --- | --- | --- |
| Atom | Position | x | y | z | Occ. | B/Å2 | *Wyckoff site* |
| Br | Br1 | 0.25 | 0.25 | 0.75 | 0.16(3) | 1.67(9) | 4*d* |
| Br | Br2 | 0 | 0 | 1 | 0.84(3) | 3.95(9) | 4*a* |
| S | S1 | 0 | 0 | 1 | 0.16(3) | 3.95(9) | 4*a* |
| S | S2 | 0.25 | 0.25 | 0.75 | 0.84(3) | 1.67(9) | 4*d* |
| S | S3 | 0.1240(3) | -0.1240(3) | 0.6240(3) | 1 | 1.83(9) | 16*e* |
| As | As1 | 0 | 0 | 0.5 | 0.5 | 1.05(5) | 4*b* |
| Ge | Ge1 | 0 | 0 | 0.5 | 0.5 | 1.05(5) | 4*b* |
| Li | T5 | 0.3064(8) | 0.0232(8) | 0.6936(8) | 0.480(8) | 3.5(2) | 48*h* |
| Li | T2 | 0.285(2) | 0.444(2) | 0.944(2) | 0.0561(9) | 3.5(2) | 48*h* |
| Li | T4 | 0.147(5) | 0.147(5) | 0.147(5) | 0.10(2) | 3.5(2) | 16*e* |

**Calculation of ionic conductivity using the from PFG-NMR data and Haven ratio**

The ionic conductivity from the PFG-NMR diffusion coefficient was calculated using the Nernst-Einstein equation^12^:

σ_NMR_ = $\frac{{cq}^{2}D^{*}}{K_{B}T}$

where D* is the Li self-diffusion coefficient obtained from PFG-NMR, q is the elementary charge of Li^+^, k_B_ is the Boltzmann constant, T is the temperature, and c is the number density of mobile charge carriers. For the argyrodite structure, 4 formula units are present per cubic unit cell. Commonly used approach for argyrodite-type lithium conductors, only one-sixth of the Li ions were considered as effective long-range charge carriers because the intra-cage Li jumps do not directly contribute to long-range inter-cage transport. Accordingly we calculated the the carrier concentration at T = 300 K. The Haven ratio was calculated according to^12^:

H_R_= σ_NMR_/σ_EIS_

**Table S7:** σ_EIS_ is the ionic conductivity measured by electrochemical impedance spectroscopy. The calculated values are summarized below.

| **x** | **a / Å** | **D* / m² s⁻¹** | **σ_NMR_ / mS cm⁻¹** | **σ_EIS_ / mS cm⁻¹** | **H_R_** |
| --- | --- | --- | --- | --- | --- |
| 0.0 | 10.1049 | 2.30 × 10^⁻12^ | 0.55 | 2.07 | 0.27 |
| 0.2 | 10.1301 | 5.08 × 10^⁻12^ | 1.25 | 6.11 | 0.20 |
| 0.3 | 10.1385 | 5.85 × 10^⁻12^ | 1.46 | 8.28 | 0.18 |
| 0.4 | 10.15211 | 8.43 × 10^⁻12^ | 2.13 | 11.00 | 0.19 |
| 0.5 | 10.15823 | 1.00 × 10^⁻11^ | 2.56 | 12.70 | 0.20 |
| 0.6 | 10.15823 | 5.99 × 10^⁻12^ | 1.56 | 8.00 | 0.19 |

Note: For x = 0.6, the lattice parameter of x = 0.5 was used for the calculation because the x = 0.6 sample contains a higher amount of impurity phases, 2 wt.% LiBr and 14 wt.% Li_4−x_Ge_1−x_As_x_S_4_.

The PFG-NMR-derived conductivity follows the same trend as the EIS conductivity, increasing with Ge substitution up to x = 0.5. The calculated Haven ratios are in the range of approximately 0.18–0.27, indicating correlated Li-ion transport in the Li_6+x_As_1−x_Ge_x_S_5_Br series. These values are comparable to those reported in a recent study on Li_6−x_PS_5−x_Br_1+x_, where the Haven ratio decreased slightly with increasing substitution and reached approximately^12^ 0.23 at x = 0.5. A Haven ratio of H_R_ = 1 corresponds to purely random, uncorrelated ion motion, whereas lower values indicate correlated ion hopping. Therefore, the obtained HR values suggest that Li-ion transport in the present argyrodite series is governed by correlated hopping, likely involving cooperative Li-ion motion and vacancy-mediated transport pathways.

***Figure S12.*** *The impedance response of the Li_6+x_As_1-x_Ge_x_S_5_Br series for x=0.0, x=0.2, x=0.3, x =0.4, x=0.5, and x=0.6.*

***Figure S13.*** *(a) Li⁺ ion mobility in the lithium argyrodite Li_6+x_As₁₋ₓGeₓS₅Br, Li_6-x_PS_5-x_Br_1+x_ and Li_6-x_AsS_5-x_Br_1+x_ series. (b) Meyer-Neldel plot illustrating log (σ_0_) versus germanium content and bromide in arsenic and phosphorus series, with (b) showing the enthalpy-entropy compensation of the pre-factor with the activation barrier. The data for Li_6-x_PS_5-x_Br_1+x_ and Li_6-x_AsS_5-x_Br_1+x_ are taken from ref^10,11^.*

***Figure S14.*** *Lithium distribution and diffusion properties. a) Li occupation extracted from AIMD simulations. b) Average lithium distances to 4d (average radius of 4d-cage) and 4a (average radius of 4a-cage) sites. c) Lithium mean squared displacement (MSD) extracted from AIMD simulations. d) Example of an individual lithium cage centered at the 4d site shown in XY projection, coupled with the distribution of distances between lithium and the cage center for Li_6_AsS_5_Br. e) Example of an individual lithium cage centered at the 4a site shown in XY projection, coupled with the distribution of distances between lithium and the cage center for Li_6_AsS_5_Br.*

***Figure S15.*** *The Raman spectral of the prepared (a) Li_6.5_As_0.5_Ge_0.5_S_5_Br, (b) Li_6_AsS_5_Br, (c) Li_5.5_PS_4.5_Br_1.5_, and (d) Li_6_PS_5_Br solid electrolytes before and after exposure in the wet air with 20% relative humidity for 10, 30 and 60 min, respectively.*

Due to the electrochemical instability of the Li_6.5_As_0.5_Ge_0.5_S_5_Br electrolyte against a lithium metal anode, a Li–In alloy (with a potential of 0.62 V vs Li⁺/Li) was employed as the anode material to mitigate interfacial side reactions and ensure stable cell operation. From the Cyclic voltammetry curve of the InLi/In | Li_6.5_As_0.5_Ge_0.5_S_5_Br | Li_6.5_As_0.5_Ge_0.5_S_5_Br-VGCF cell (Figure S1), it can be seen that the reduction potential of the Li_6.5_As_0.5_Ge_0.5_S_5_Br electrolyte is 1.06 V (*vs*. LiIn/Li) against the LiIn/In anode, corresponding to 1.68 V (*vs*. Li^+^/Li) against the LiIn/In anode.

***Figure S16***. The CV curve of the InLi/In | Li_6.5_As_0.5_Ge_0.5_S_5_Br | Li_6.5_As_0.5_Ge_0.5_S_5_Br-VGCF cell.

***Figure S17.*** *Voltage profiles of In/InLi| Li_6.5_As_0.5_Ge_0.5_S_5_Br |s-NCM90- Li_6.5_As_0.5_Ge_0.5_S_5_Br -C65 cells with 8.83 mg cm^−2^ cathode active loading at different cycles. (a) at 0.1C, (b) at 1C, and (d) at 2C. (c) Cycling performance of Li_6.5_As_0.5_Ge_0.5_S_5_Br cells at 2C.*

**References:**

(1)	Perdew, J. P.; Burke, K.; Ernzerhof, M. Generalized Gradient Approximation Made Simple. *Phys. Rev. Lett.* **1996**, *77*, 3865–3868.

(2)	Perdew, J. P.; Ruzsinszky, A.; Csonka, G. I.; Vydrov, O. A.; Scuseria, G. E.; Constantin, L. A.; Zhou, X.; Burke, K. Restoring the Density-Gradient Expansion for Exchange in Solids and Surfaces. *Phys. Rev. Lett.* **2008**, *100*, 136406.

(3)	Kresse, G.; Furthmüller, J. Efficiency of Ab-Initio Total Energy Calculations for Metals and Semiconductors Using a Plane-Wave Basis Set. *Comput. Mater. Sci.* **1996**, *6*, 15–50.

(4)	Blöchl, P. E. Projector Augmented-Wave Method. *Phys. Rev. B* **1994**, *50*, 17953–17979.

(5)	Nosé, S. A Unified Formulation of the Constant Temperature Molecular Dynamics Methods. *J. Chem. Phys.* **1984**, *81*, 511–519.

(6)	Hoover, W. G. Canonical Dynamics: Equilibrium Phase-Space Distributions. *Phys. Rev. A.* **1985**, *31*, 1695–1697.

(7)	de Klerk, N. J. J.; van der Maas, E.; Wagemaker, M. Analysis of Diffusion in Solid-State Electrolytes through MD Simulations, Improvement of the Li-Ion Conductivity in β-Li3PS4 as an Example. *ACS Appl. Energy Mater.* **2018**, *1*, 3230–3242.

(8)	Azizi, V.; Smeets, S.; Lavrinenko, A. K.; Ciarella, S.; Famprikis, T. *gemdat (1.2.1). Zenodo*. https://doi.org/10.5281/zenodo.8401669.

(9)	Kong, S.; Deiseroth, H.; Reiner, C.; Gün, Ö.; Neumann, E.; Ritter, C.; Zahn, D. Lithium Argyrodites with Phosphorus and Arsenic: Order and Disorder of Lithium Atoms, Crystal Chemistry, and Phase Transitions. *Chemistry – A European Journal* **2010**, *16*, 2198–2206.

(10)	Gautam, A.; Al-Kutubi, H.; Famprikis, T.; Ganapathy, S.; Wagemaker, M. Exploring the Relationship Between Halide Substitution, Structural Disorder, and Lithium Distribution in Lithium Argyrodites (Li6–xPS5–xBr1+ x). *Chemistry of Materials,* **2023,** 35, 8081-8091.

(11)	Zhou, R.; Gautam, A.; Suard, E.; Li, S.; Ganapathy, S.; Chen, K.; Zhang, X.; Nan, C.-W.; Wang, S.; Wagemaker, M. Boosting Ionic Conductivity and Air Stability in Bromide-Rich Thioarsenate Argyrodite Solid Electrolytes. Advanced Functional Materials **2025**, 35, 2420971

(12)	Adeli, P.; Bazak, J. D.; Park, K. H.; Kochetkov, I.; Huq, A.; Goward, G. R.; Nazar, L. F. Boosting Solid-State Diffusivity and Conductivity in Lithium Superionic Argyrodites by Halide Substitution. *Angew. Chem. Int. Ed.* **2019**, *58*, 8681–8686.
